# Differential Protein Expression Profiles of Cyst Fluid from Papillary Thyroid Carcinoma and Benign Thyroid Lesions

**DOI:** 10.1371/journal.pone.0126472

**Published:** 2015-05-15

**Authors:** Andrii Dinets, Maria Pernemalm, Hanna Kjellin, Vitalijs Sviatoha, Anastasios Sofiadis, C. Christofer Juhlin, Jan Zedenius, Catharina Larsson, Janne Lehtiö, Anders Höög

**Affiliations:** 1 Department of Oncology-Pathology, Karolinska Institutet, SE-171 76, Stockholm, Sweden; 2 Cancer Center Karolinska, Karolinska University Hospital, SE-171 76, Stockholm, Sweden; 3 Department of Molecular Medicine and Surgery, Karolinska Institutet, SE-171 76, Stockholm, Sweden; 4 Cancer Proteomics Mass Spectrometry, Science for Life Laboratory, SE-171 65, Stockholm, Sweden; 5 Department of Pathology-Cytology, Karolinska University Hospital, SE-171 76, Stockholm, Sweden; Universite Libre de Bruxelles (ULB), BELGIUM

## Abstract

Cystic papillary thyroid carcinoma (cPTC) is a subgroup of PTC presenting a diagnostic challenge at fine needle aspiration biopsy (FNAB). To further investigate this entity we aimed to characterize protein profiles of cyst fluids from cPTC and benign thyroid cystic lesions. In total, 20 cPTCs and 56 benign thyroid cystic lesions were studied. Profiling by liquid chromatography tandem mass spectrometry (LC-MS/MS) was performed on cyst fluids from a subset of cases after depletion, and selected proteins were further analyzed by Western blot (WB), immunohistochemistry (IHC) and enzyme-linked immunosorbent assay (ELISA). A total of 1,581 proteins were detected in cyst fluids, of which 841 were quantified in all samples using LC-MS/MS. Proteins with different expression levels between cPTCs and benign lesions were identified by univariate analysis (41 proteins) and multivariate analysis (59 proteins in an orthogonal partial least squares model). WB analyses of cyst fluid and IHC on corresponding tissue samples confirmed a significant up-regulation of cytokeratin 19 (CK-19/CYFRA 21-1) and S100A13 in cPTC vs. benign lesions. These findings were further confirmed by ELISA in an extended material of non-depleted cyst fluids from cPTCs (n = 17) and benign lesions (n = 55) (p<0.05). Applying a cut-off at >55 ng/ml for CK-19 resulted in 82% specificity and sensitivity. For S100A13 a cut-off at >230 pg/ml revealed a 94% sensitivity, but only 35% specificity. This is the first comprehensive catalogue of the protein content in fluid from thyroid cysts. The up-regulations of CK-19 and S100A13 suggest their possible use in FNAB based preoperative diagnostics of cystic thyroid lesions.

## Introduction

Papillary thyroid carcinoma (PTC) is the most common form of thyroid malignancy. The tumor is usually well-differentiated and is characterized by typical cytological and histopathological features [1]. The cystic variant of PTC (cPTC) constitutes 4–13% of all PTCs [2, 3]. It exhibits classical histopathological features of PTC, but different morphological properties due to its presentation as a mural nodule within a thyroid cyst. The diagnosis is challenging, since the fine needle aspiration biopsy (FNAB) of a cystic thyroid nodule usually results in a relatively large volume of cyst fluid, but may contain insufficient amounts of representative tumor cells. This may lead to cytological reports in which malignancy is not recognized [4]. Attempts have been made to identify diagnostic markers for thyroid cystic lesions by using biochemical, immunohistochemical or genetic markers [4–6]. The most frequently used immunohistochemical markers to confirm a PTC diagnosis are cytokeratin 19 (CK-19) and Hector Battifora mesothelial antigen-1 (HBME-1) [6, 7]. Although these proteins are commonly up-regulated in PTC, they can also be expressed in benign thyroid lesions, leading to difficulties in establishing the preoperative diagnosis [8]. Routine cytological investigation is performed mainly on the cellular component of the FNAB specimen, and less attention is given to the fluid component [3, 5, 6, 9, 10]. Identification of novel diagnostic markers to distinguish cPTC from benign cystic thyroid lesions is warranted and could be based on protein-based screening approaches.

Proteomic techniques are increasingly applied in the search for diagnostic and prognostic markers in cancer [11–18]. In thyroid cancer, protein expression patterns have been evaluated by these techniques with the aim of distinguishing benign and malignant thyroid tumors [11–13, 17, 19–21]. Despite harboring various products of the epithelial cells’ activity, the liquid component of a thyroid cyst is routinely discarded in FNAB workflow. By contrast, in ovarian cancer and hemangioblastoma, the protein content of cyst fluids is frequently investigated regarding their protein content [22, 23]. Given the possible diagnostic utility of the thyroid cyst fluid content which could improve FNAB, this study aimed at identifying differentially expressed proteins in cPTC by applying a proteomic approach.

## Material and Methods

### Ethical Statement

All samples were collected and stored at the Karolinska Biobank. Prior to surgical operations or other sampling of tissues at the Karolinska University Hospital, all patients are routinely asked for permission to save their tissues or biological fluids for research purposes. All patients are informed both verbally and by a written patient information. Patient consent (or denial) is obtained verbally and thereafter documented in the patients’ medical records as a standard procedure. The procedure of obtaining verbal consent from patients is in agreement with Swedish law and ethical regulations. The research project as well as the procedure of obtaining patient consent for biobanking were approved by the local ethical committee, at the time called The Karolinska Institutet Research Ethical Committee (permission number 03–517).

### Patients and samples

Seventy-six cases of thyroid cystic lesions were collected for the study, including 56 benign lesions (51 nodular colloid goiters and 5 follicular thyroid adenomas) and 20 cPTCs (Table 1). Patients were surgically treated within the period 2011–2013 at the Department of Breast and Endocrine Surgery at Karolinska University Hospital, Stockholm, Sweden. Samples of cyst fluid were collected by aspiration from the post-operative specimens. All aspirations were performed by an experienced pathologist or technician and using a syringe with a needle corresponding to the routine FNAB.

**Table 1:** Summary of clinical and histopathological features for all cystic PTC (cPTC) and benign cystic lesions in the study.

Parameter	cPTCs (n = 20)	Benign cystic lesions (n = 56)	cPTC vs. benign p-value
***Results from***			-
LC-MS/MS and Western blot analysis	6	7	
IHC	7	7	
ELISA	17	55	
***Age at diagnosis***			0.4
Mean (range) years	45 (24–73)	50 (21–81)	
***Gender***			0.2
Male	11 (55%)	21 (38%)	
Female	9 (45%)	35 (62%)	
Female: Male ratio	1: 0.8	1: 1.7	
***Preoperative diagnosis at FNAB***			0.4
PTC	8 (47%)	0	
Suspicious	5 (29%)	0	
Follicular tumor	0	6 (12%)	
Benign	4 (22%)	46 (88%)	
Not available	3	4	
***Postoperative diagnosis***			-
PTC	20 (100%)	0	
Nodular colloid goiter	0	51 (91%)	
FTA	0	5 (9%)	
***Location of primary tumor / lesion***			
Right lobe	14 (70%)	28 (49%)	0.2
Left lobe	5 (25%)	27 (47%)	0.1
Isthmus	1 (5%)	1 (4%)	0.5
***Type of surgery***			
Total thyroidectomy	17 (85%)	13 (23%)	<0.001
Hemithyroidectomy	3 (15%)	42 (75%)	<0.001
Isthmus resection	0	1 (2%)	1
***Size of cyst***			
Mean (range) cm	2.8 (0.5–6)	3.5 (0.5–8)	0.14
***Size of PTC***			
Mean (range) cm	3 (1.3–6)	-	
***Spread PTC***			
Metastases to local lymph nodes	12 (60%)	-	
Extrathyroidal extension	3 (15%)	-	
***Bilateral or multifocal PTC***			
Multifocal	9 (45%)	-	
Bilateral PTC	7 (35%)	-	
***MIB-1 proliferation index***			
Mean (range) %	2.3 (0.5–6)	-	
***Thyroglobulin expression***			
Positive cells (range) %	80 (50–100)	-	

F = female; M = male; FNAB = fine needle aspiration biopsy; PTC = papillary thyroid carcinoma; FTA = Follicular thyroid adenoma; "-" = not applicable

The diagnosis was confirmed by histopathological examination of sections routinely stained with hematoxylin and eosin (H&E) according to the WHO classification of thyroid tumors [1]. Clinical information was retrieved for all cases from cytology and pathology reports and medical records. MIB-1 proliferation index was determined by routine immunohistochemistry (IHC) using the MIB-1 antibody against the Ki-67 antigen. Expression of thyroglobulin was assessed by IHC in the routine clinical setting. For cPTCs, the tumor size was determined as equal to the diameter of the cyst (for intracystic tumors) or as a sum of the cyst and the extracystic tumor component (for tumors with additional tumor growth outside the cyst).

A subset of 7 cPTCs and 7 benign cystic thyroid lesions were included in the protein profiling and verification by Western blot analysis (Table 1, S1 Table). Case cPTC-5 was excluded from the data analysis, as a colloid goiter cyst was detected adjacent to the PTC, and therefore the integrity of the cyst fluid as PTC-related only could not be guaranteed. Formalin-fixed paraffin embedded (FFPE) tissue samples were obtained for all 14 cases and used for IHC. In addition four of the cPTCs (cases 1, 4, 6, and 7) and 6 of the benign cystic lesions (Benign-2 excluded due to lack of fluid samples) from the LC-MS/MS screening were included in the ELISA analyses together with additionally 63 cases of cystic thyroid lesions (49 benign lesions and 13 cPTCs) (Table 1).

Additionally, cyst fluids and tumor tissues were collected from other types of cystic tumors including: one parathyroid adenoma (PHPT), two follicular thyroid carcinomas (FTC), two toxic follicular thyroid adenomas (FToxA), one anaplastic thyroid carcinoma (ATC) and two lymph node metastases of cPTC (S2 Table). These samples served as references for Western blot analysis (PHPT and ATC only), ELISA, and IHC together with specimens of normal pancreas from anonymized patients.

### Handling of cyst fluids

To separate cells and aggregates from the cyst fluid, all samples were centrifuged twice at 2,500 rpm for 10 min at room temperature (RT). Measurement of total protein concentration was performed by using a spectrophotometer ND-1000 (NanoDrop Technologies, USA). Protein concentrations are given in S3 Table together with information about viscosity and appearance of cyst fluids determined by visual inspection.

### Depletion of high abundant proteins

To remove high abundant proteins (albumin, haptoglobin, IgA, IgG, transferrin, antitrypsin), a depletion procedure was performed by using the Multi Agilent Affinity Removal Column (Hu-6, 4x 100 mm Agilent Technologies, USA) on an ÄKTA chromatography system (GE Healthcare) according to the manufacturer’s instructions. The depleted fractions were concentrated in a 5 kDa cut-off filter (Vivaproducts, Inc, USA).

### iTRAQ labeling and digestion

Seventy μg of each depleted sample was dried using a SpeedVac system, dissolved in iTRAQ dissolution buffer and digested with trypsin. The digested samples were labeled with iTRAQ Reagents (SCIEX, USA) and subsequently pooled into two tubes (S3 Table). The pooled and iTRAQ labeled samples were purified using two 1 ml Strata X-C 33 μm columns (Phenomenex, Inc.) applying previously described protocols [15, 16, 24].

### Isoelectric focusing and extraction

After the strong-cation exchange clean-up, the iTRAQ labeled samples underwent isoelectric focusing (IEF) by using two 24 cm 3.7–4.9 immobilized pH gradient (IPG) strips (GE Healthcare). The samples were rehydrated in 8 M urea with bromophenol blue and 1% IPG buffer, subsequently loaded to the IPG strip and run according to previously published protocols [15, 16, 24]. After IEF, the IPG strip was fractionated to 72 fractions. The obtained fractions were dried using SpeedVac and frozen at -20°C until analysis.

### Liquid chromatography tandem mass spectrometry (LC-MS/MS)

LC-MS/MS was performed essentially according to previously published methodology [15, 16]. Before the analysis fractions were dissolved in 8 μl of 3% ACN with 0.1% formic acid. Three μl from each fraction was injected into online HPLC-MS performed on a hybrid LTQ-Orbitrap Velos mass spectrometer (Thermo Scientific). An Agilent HPLC 1200 system (Agilent Technologies) was used to provide the 50 min gradient for online reversed-phase nano-LC at a flow of 0.4 μl/min. Each sample was injected into a C18 guard desalting column (Agilent Technologies) before entering into a 15 cm long C18 picofrit column with a 100 μm internal diameter and 5 μm bead size (Nikkyo Technos Co., Japan) installed on to the nano electrospray ionization (NSI) source. Precursors were isolated with a 2 m/z width and dynamic exclusion was used with 60 sec duration. We enabled “preview mode” for FTMS master scans, which proceeded at 30,000 resolution (profile mode). Data-dependent MS/MS (centroid mode) followed in two stages: firstly, the top 5 ions from the master scan were selected for collision-induced dissociation (CID, at 35% energy) with detection in the ion trap (ITMS). Then, the same 5 ions underwent higher energy collision dissociation (HCD, at 37.5% energy) with detection in the Orbitrap (FTMS). The entire duty cycle lasted ~3.5 sec.

The MS/MS data was searched using Sequest under the software platform Proteome Discoverer 1.3.0.339 (Thermo Scientific) against the Swissprot_human_20121008.fasta protein sequence database using one 99% peptide confidence as cut-off. A precursor mass tolerance of 15 ppm, and product mass tolerances of 0.02 Da for HCD-FTMS and 0.8 Da for CID-ITMS were used. Further used settings were: trypsin with 1 missed cleavage; IAA on cysteine as fixed modification and iTRAQ 8plex on lysine and N-terminal and oxidation of methionine as variable modification and phosphorylation of serine, threonine or tyrosine as variable modifications. Probabilities for phosphosite localization (within each phosphopeptide) were calculated using the phosphoRS node of Proteome Discoverer. Quantitation of iTRAQ 8plex reporter ions was performed by Proteome Discoverer on HCD-FTMS tandem mass spectra using an integration window tolerance of 20 ppm and false discovery rate was estimated using percolator (part of PD 1.3) for false discovery rate cut-off. The LC-MS/MS data was deposited to the ProteomeXchange Consortium [25] via the PRIDE partner repository (http://www.proteomecentral.proteomexchange.org) with the dataset identifier PXD000996.

### Immunohistochemistry (IHC)

IHC was performed for all 14 samples used in the LC-MS/MS screening as well as for other cystic thyroid and parathyroid reference tissue samples (S1 and S2 Tables) according to previously published protocols [11, 12, 26, 27]. Briefly, 4 μm FFPE tissue sections were deparaffinized in xylene, rehydrated in graded series of ethanol and incubated in citrate buffer (Dako, Sweden) for 20 min at 95–99°C for antigen retrieval. Slides were incubated in 0.5% H_2_O_2_ to deactivate endogenous peroxidase. Unspecific binding sites were blocked by 1% bovine serum albumin. Slides were incubated overnight with a primary antibody: polyclonal rabbit anti-S100A13 (HPA019592, Atlas Antibodies AB, Sweden) at dilution 1:1,500; anti-annexin A3 (HPA013398, Atlas Antibodies AB) at 1:1,500; polyclonal rabbit anti-CMBL (HPA036571, Atlas Antibodies AB) at 1:1,000; monoclonal mouse anti-CK-19 (clone RCK 108, Dako) at 1:300; or anti-human mesothelial cell (clone HBME-1, Dako) at 1:400. A secondary biotinylated antibody was applied followed by an avidin-biotin-complex (ABC) conjugated to horseradish peroxidase (HRP) according to the manufacturer’s instructions (Vectastain, Vector Burlingame). The result was visualized with diaminobenzidine and counterstaining in hematoxylin.

Microscopical evaluation of IHC slides was performed according to previously described protocols and equipment [11, 12, 26, 27]. A semi-quantitative scoring system was applied to evaluate the intensity of cytoplasmic staining as weak (1), weak-to-moderate (1.5), moderate (2), moderate-to-strong (2.5) or strong (3). In addition the proportions of positive tumor cells were estimated for each target protein and presented in percentage.

### Western blot analyses

Western blot was performed on the depleted cyst fluids from 13 cases analyzed by LC-MS/MS. In addition depleted cyst fluids of cystic PHPT and ATC and total protein extract from the lung adenocarcinoma cell line A549 were included as references. Proteins were separated in a 10-well 12% NuPage Bis-Tris gel and subsequently blotted to nitrocellulose membrane with 0.45 μm pores (Life Technologies, USA) [11, 12]. The membranes were incubated in 5% non-fat milk solution for 1 h at RT followed by overnight incubation at 4°C with primary antibodies: monoclonal mouse anti-CK-19 (sc-6278, Santa Cruz Biotechnology, Inc., USA), polyclonal rabbit anti-S100A13 (HPA019592, Atlas Antibodies AB) at 1:100; anti-annexin A3 (HPA013398, Atlas Antibodies AB) at 1:100 or anti-CMBL (HPA036571, Atlas Antibodies AB) at 1:250. Membranes were then incubated for 1 h at RT with an HRP-conjugated secondary antibody: goat anti-rabbit at dilution 1:8,000 or anti-mouse at dilution 1:10,000. After application of chemiluminescent substrates for detection of HRP (Life Technologies), membranes were scanned using a luminescent image analyzer LAS-1000plus and protein bands were documented using a LAS-1000 image reader (Fuji Photo Film, Japan).

### Enzyme-linked immunosorbent assay (ELISA)

A quantitative sandwich ELISA was performed according to the protocols of the manufacturers on non-depleted cyst fluid samples of 72 thyroid lesions (17 cPTCs and 55 benign lesions) and the cystic tissue reference samples. The calibrators and samples were loaded to 96-well microplate in duplicate and bound by the immobilized primary antibody using assays for: CK-19/CYFRA 21–1 (KA4024, Abnova, Taiwan), S100A13 (E01S0025, Bluegene; DY4327 R&D systems, Abingdon, UK), and vimentin (KA3127, Abnova). After washings, an HRP-conjugated antibody was added to the samples. The optical density (O.D.) was determined using a VERSA max microplate reader by photometry at 450 nm and documented by SOFTmax PRO v4 (Molecular Devices, USA), and protein concentrations were interpolated from the O.D. data using a standard curve.

### Statistical analyses

Statistical analyses were performed using the data analysis software system Statistica v12 (StatSoft Scandinavia AB, Sweden). To compare findings in the studied groups we used parametric and non-parametric univariate tests (Student’s t-test, Mann-Whitney U Test). Receiver operating characteristics (ROC) curve test was performed to determine optimal cut-off values for target proteins by analyses of area under ROC curve (AUC), sensitivity and specificity, positive predictive value (PPV), negative predictive value (NPV), positive likelihood ratio (PLR), negative likelihood (NLR); diagnostic odds ratio (DOR) was calculated as suggested by Glas *et al*. [28]. The software SIMCA (SIMCA-P+ 12.0, Umetrics, Sweden) was used for multivariate statistics and modeling. The multivariate analysis was performed on mean centered, unit variance scaled data, assuming equal importance of each protein. Orthogonal partial least squares (OPLS) were used to build classification models. Variable Importance in projection value (VIP) was used for optimization of OPLS models and for evaluation of protein importance. The OPLS models were validated by sevenfold cross validation. Proteins with significant VIP throughout the cross validation of the model were selected for the optimized model. CV-ANOVA was used to judge the model validity. Results with p-values <0.05 were considered as statistically significant.

Hierarchical clustering was performed using Genesis software (http://www.genome.tugraz.at/genesisclient/genesisclient_description.shtml). Ingenuity Pathway Analysis (IPA) was used for pathway analysis (Ingenuity Systems, http://www.ingenuity.com). In brief, Ingenuity uses Fisher’s exact test to calculate the probability that a set of proteins is associated to a pathway by chance. P-values <0.05 were taken as significant.

The gene ontology (GO) enrichment analysis was performed using Gorilla [29] and the network analysis of the GO terms was performed using Revigo [30].

## Results

### Identification of cystic PTC (cPTC) cases

**Fig 1:**
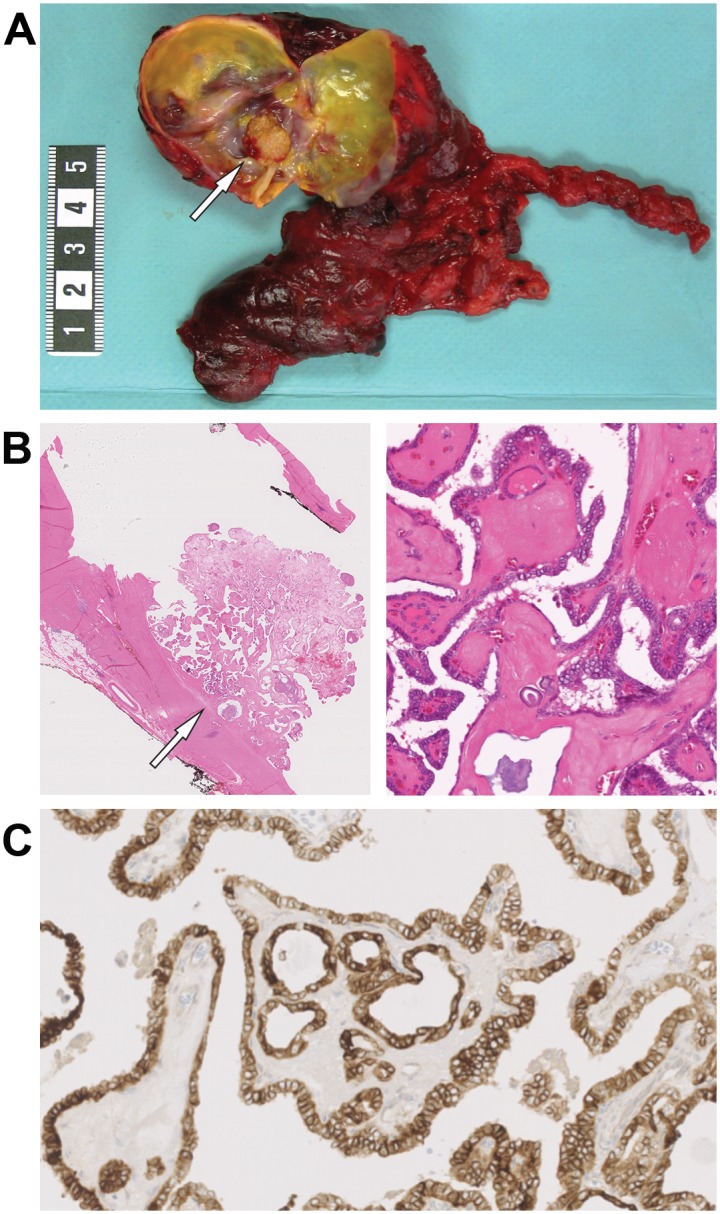
Macroscopical and histopathological features of a cPTC. (**A**) A mural nodule diagnosed as a cPTC (arrow) in the right lobe of a resected thyroid specimen. (**B**) Histopathological slide illustrating the same mural nodule (arrow) after H&E staining exhibiting histopathological features of PTC, spots of calcifications and partially fibrous capsule of thyroid cyst. The sample is shown in x7 (left) and x110 (right) magnifications. (**C**) Immunohistochemistry showing expression of thyroglobulin in cPTC tumor cells (magnification x40).

We identified a total of 227 patients who were diagnosed with PTC and operated on at Karolinska University Hospital within a three-year period from January 2011 to December 2013. Twenty of these 227 cases were classified as cPTC giving a proportion of 9% cPTCs in this study cohort (Table 1). A representative case of cPTC is shown in Fig 1.

The 20 cPTCs were subsequently analyzed together with 56 benign cystic thyroid lesions (Table 1). These cases were identified after analyses of the FNAB reports (n = 69) or by identification of cystic lesion in surgically excised thyroid tumors in other samples (n = 7). A preoperative diagnosis of PTC was established in 8 cases (47%), whereas 9 cPTC cases (53%) had suspicious or benign FNAB results illustrating the diagnostic challenges for preoperative identification of cPTC (Table 1). A distinct preoperative diagnosis for benign cystic thyroid lesions was established by FNAB for 46 cases (88%), whereas for 6 patients (12%) the diagnosis “follicular neoplasm” was suggested at FNAB.

### Protein profiling by LC-MS/M*S*

**Fig 2:**
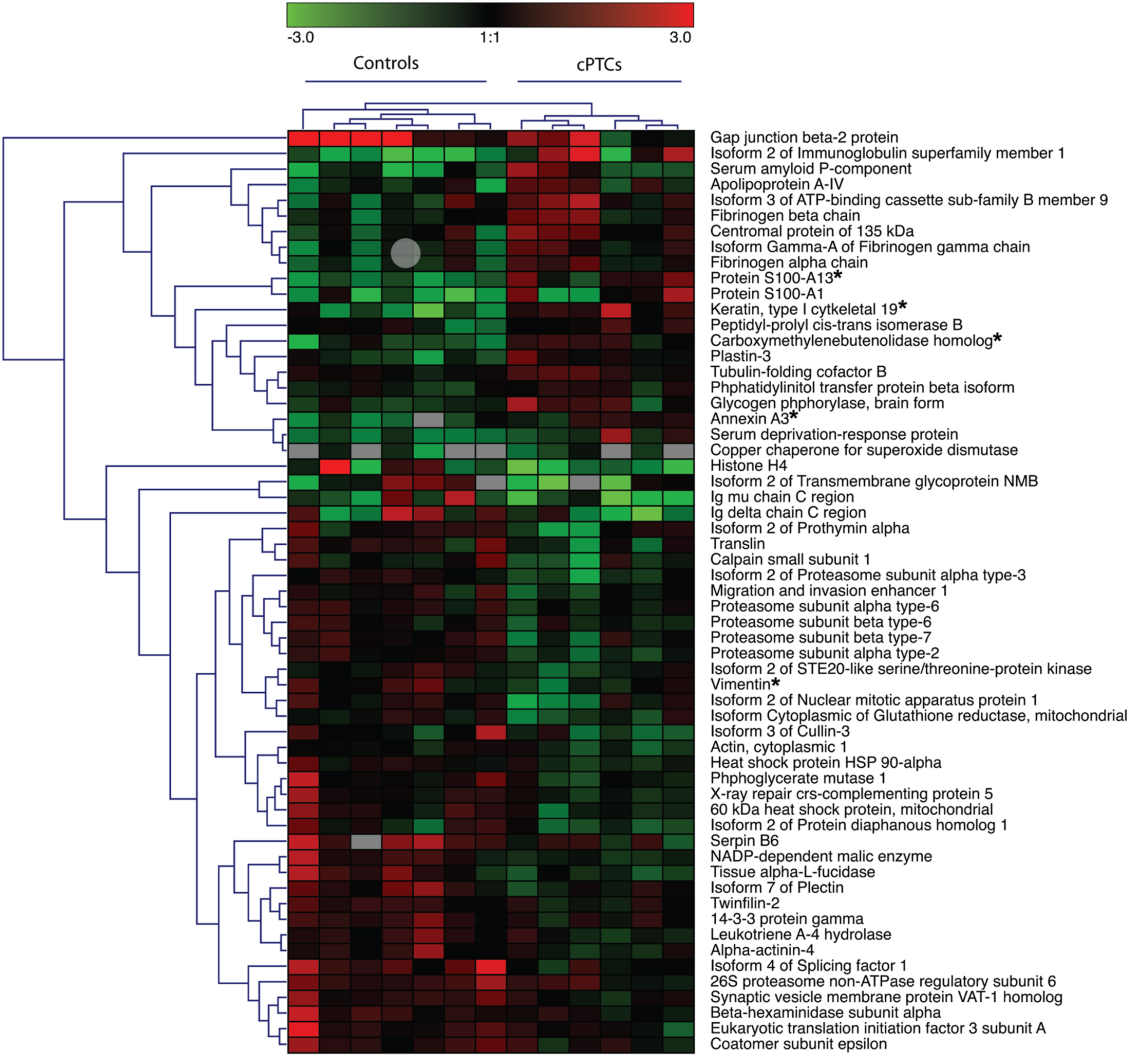
Unsupervised hierarchical clustering showing the 59 proteins in the OPLS model. Up-regulation and down-regulation of proteins in cPTC vs. benign lesions are indicated in red and green, respectively. Proteins selected for further verification are indicated by (*).

Depleted cyst fluids from cPTCs and benign cystic thyroid lesions were profiled by LC-MS/MS. In total, 1,581 proteins were identified from the cyst fluid samples using a confidence limit of one peptide at 99% (p<0.01). Of these, totally 841 proteins were quantified in all 13 samples (S4 Table), which were subsequently subjected to gene ontology (GO) enrichment analysis (S1 Fig, S5 Table). The top five enriched GO terms were “blood microparticle”, “extracellular region part”, “extracellular organelle”, “extracellular membrane-bounded organelle” and “extracellular vesicular exosome”. This indicated high similarity with plasma and/or blood samples in terms of protein content. In a comparative GO analysis with a published plasma data set [31], the cyst fluid showed high similarity with plasma, strengthening this observation (S2 Fig). To identify signaling pathways differing between the cPTCs and benign lesions we applied pathway analysis in combination with multivariate data analysis. Using multivariate OPLS analysis a highly predictive model of 59 proteins was obtained (p = 0.0027; R2Y = 0.969 and Q2 = 0.841) for distinguishing between cPTCs and benign lesions (Fig 2, S6 Table). IPA analysis of these 59 proteins identified “protein ubiquitination”, “extrinsic prothrombin activation”, “intrinsic prothrombin activation”, “the coagulation system” and “gluconeogenesis” as the top five significantly enriched pathways (p<0.01). Detailed comparison of the cPTCs and benign lesions by univariate analysis revealed significantly different expression for 41/841 proteins (p-values <0.05, Student’s t-test, S4 Table). Of these, 10 proteins were also differently expressed between the cPTCs and benign lesions using a more stringent p-value cut-off of 0.01 (Table 2), and 40 proteins were also included in the OPLS model (Fig 2, S6 Table).

**Table 2:** Top 10 proteins identified by LC-MS/MS with significantly different expression in cyst fluids from cPTCs (n = 6) compared to benign lesions (n = 7) after depletion.

Accession ID	Protein name	Gene symbol	No. of peptides		cPTC vs. Benign	
			Unique	Total	Log2 Fold Change	p-value
***Over-expressed in cPTC vs*. *Benign***						
P08727	Keratin, type I cytoskeletal 19 (*)	*KRT19*	8	61	2	0.002
Q99584	Protein S100-A13 (*)	*S100A13*	3	33	1.9	0.001
P12429	Annexin A3 (*)	*ANXA3*	3	5	1.4	0.006
Q96DG6	Carboxymethylenebutenolidase homolog (*)	*CMBL*	3	7	1.2	0.004
Q8N6C5-2	Isoform 2 of Immunoglobulin superfamily member 1	*IGSF1*	3	16	3.1	0.006
***Under-expressed in cPTC vs*. *Benign***						
P25787	Proteasome subunit alpha type-2	*PSMA2*	3	21	-0.9	0.001
P04066	Tissue alpha-L-fucosidase	*FUCA1*	3	19	-1.5	0.008
P48163	NADP-dependent malic enzyme	*ME1*	4	11	-1.3	0.008
P35237	Serpin B6	*SERPINB6*	2	6	-1.3	0.010
P25788-2	Isoform 2 of Proteasome subunit alpha type-3	*PSMA3*	2	4	-0.9	0.005

(*) Selected for further analyses

### Verification of selected proteins by IHC and Western blot analyses

Four proteins that were found up-regulated in cPTCs were selected for verification: cytokeratin 19 (CK-19), S100A13, annexin A3 (ANXA3) and carboxymethylenebutenolidase homolog (CMBL). The selection was based on the number of peptides, their identification as up-regulated in cPTCs vs. benign cystic lesions in univariate analyses (Table 2) and the OPLS model (S6 Table) as well as the availability of detection antibodies or assays. Additionally, we included HBME-1 in the IHC experiments, which is a well-known marker of PTC in routine clinical settings.

**Fig 3:**
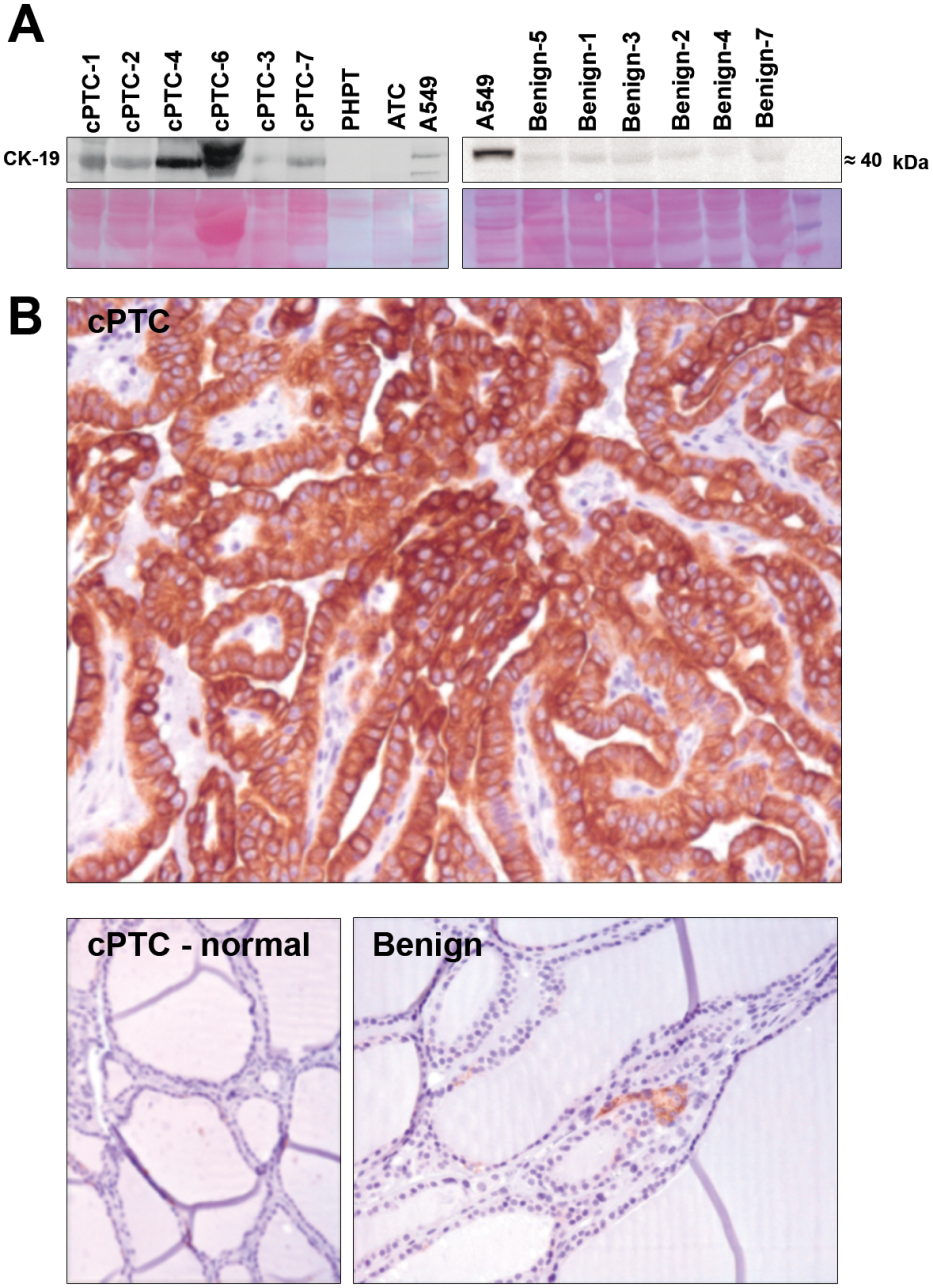
Expression of CK-19 in cPTC, benign cystic lesions and references. (A) Western blot analyses of CK-19 with depleted cyst fluids of cPTCs and benign lesions from the LC-MS/MS screening samples. Depleted cyst fluids of a parathyroid adenoma (PHPT), anaplastic thyroid carcinoma (ATC) and total protein extract from A549 cells are shown as reference. Representative transfer of proteins to nitrocellulose membrane stained with 0.1% Ponceau S solution used as loading control is shown below. (**B**) Immunohistochemistry showing CK-19 expression in cPTC tissue tumor cells but not in surrounding normal thyroid cells (cPTC—normal) or multinodular goiter (Benign).

**Fig 4:**
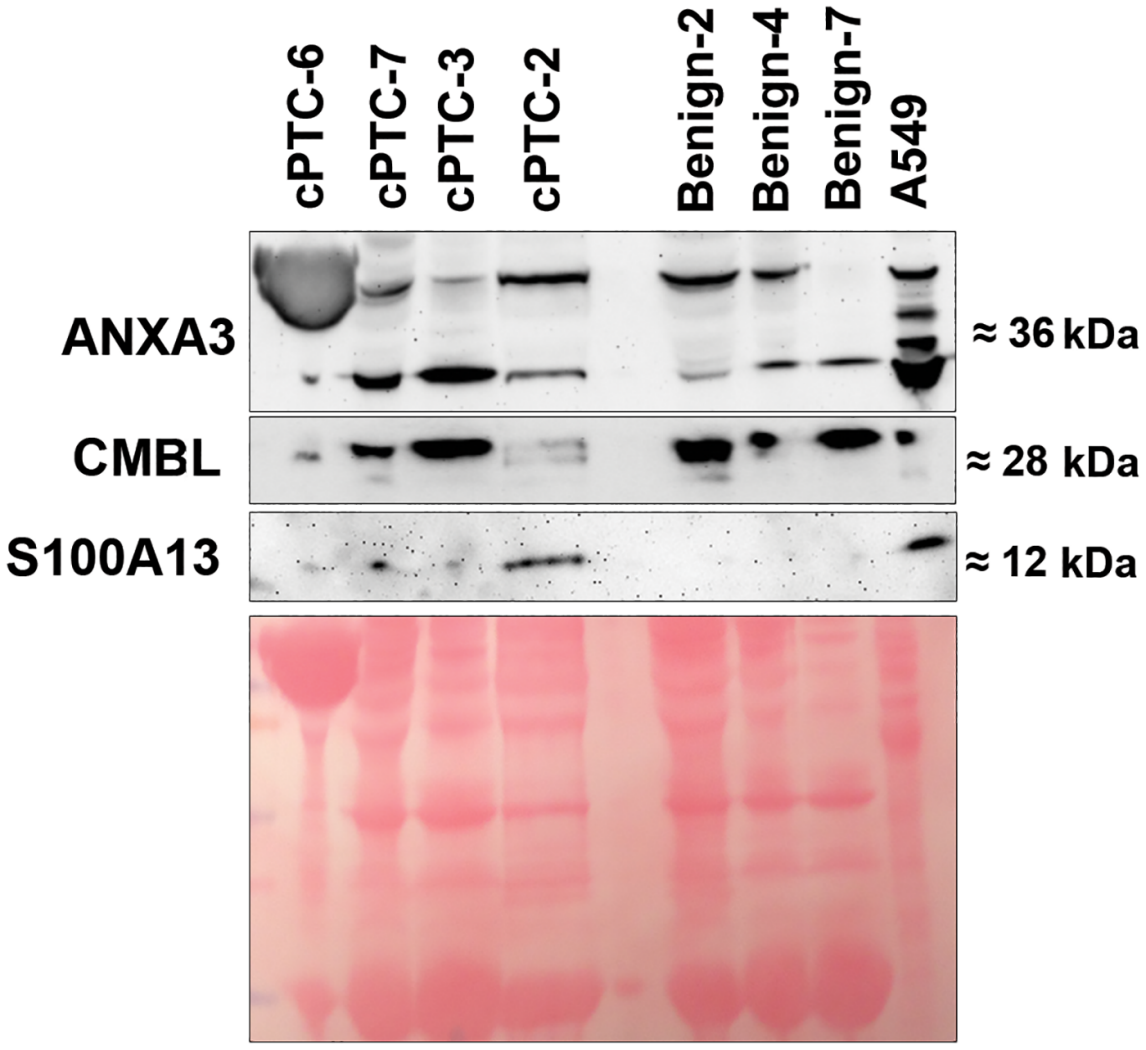
Western blot analyses of ANXA3, CMBL and S100A13 expression. Depleted cyst fluids from cPTCs and benign lesions are shown together with total protein extract from A549 cells. Protein sizes are indicated to the right in kDa. Ponceau S solution used as loading control is shown below.

Western blot analyses showed expression of expected protein products for CK-19 (Fig 3), ANXA3, CMBL and S100A13 in cyst fluids (Fig 4). CK-19 showed a higher expression in cPTCs as compared to benign thyroid cases. Furthermore, S100A13 showed weak expression in 4 (57%) of the cPTC cases, while in the remaining cPTCs and all benign samples no S100A13 expression was detected. ANXA3 and CMBL were both expressed in the cyst fluids of cPTCs and benign samples without obvious differences between the two groups.

**Fig 5:**
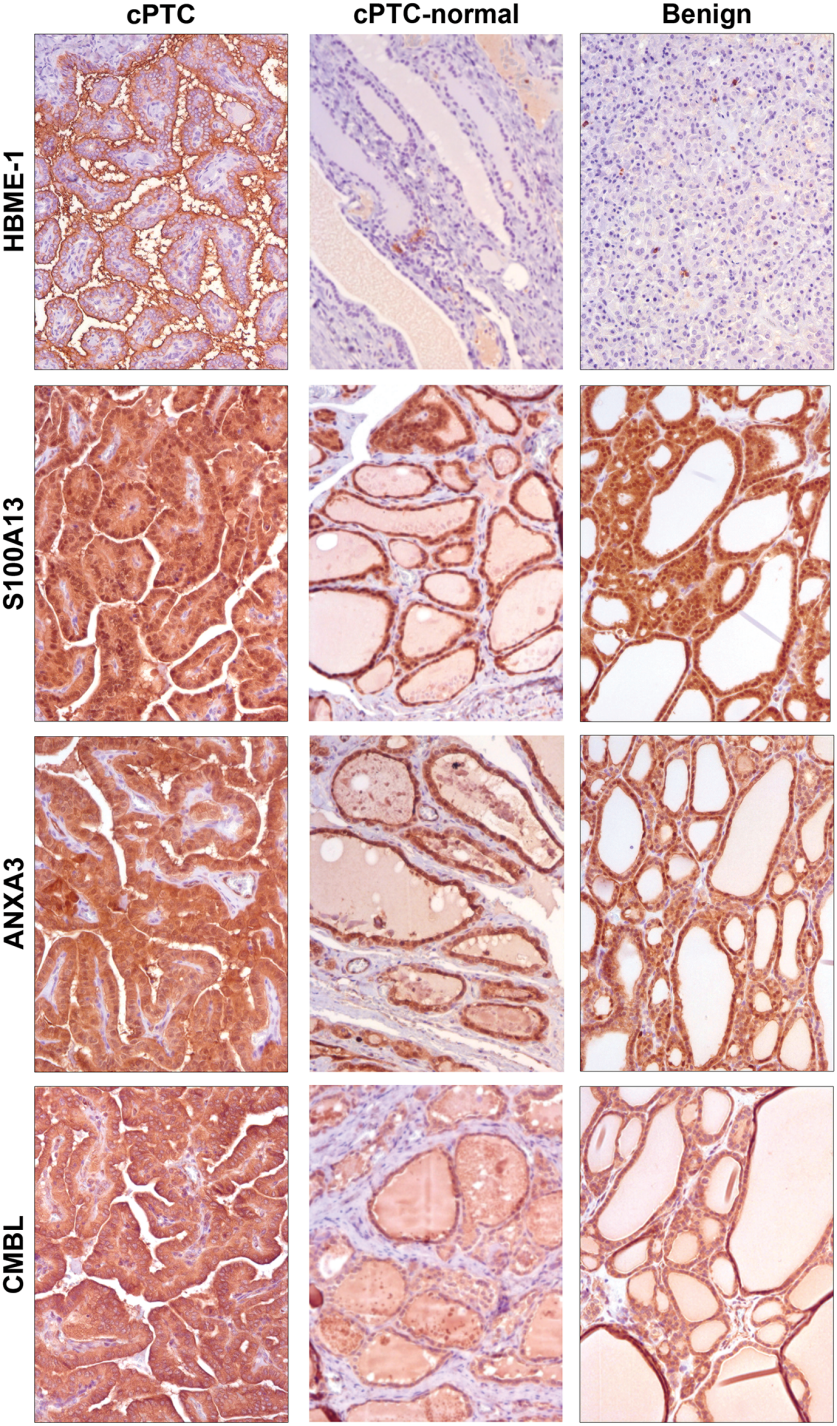
Detection of protein expressions by immunohistochemistry. Expression of HBME-1, S100A13, ANXA3, and CMBL showed in cPTC (left), normal thyroid (middle), and benign thyroid lesions (right) (magnification x60). Up-regulation of HBME-1 in 100% of cPTC cells as compared to accompanying normal thyroid cells is shown together with negative immunoexpression of HBME-1 in a follicular thyroid adenoma. Microphotographs demonstrate moderate-to-strong expressions of S100A13, ANXA3, and CMBL in cPTC, surrounding normal thyroid cell and in colloid goiter.

The main findings from IHC analyses are summarized in Table 3 and typical staining patterns are shown in Figs 3 and 5. All analyzed proteins were expressed in cPTCs. HBME-1 revealed higher expression in all cPTCs as compared to benign lesions (Fig 5) in which only single positive cells showed a weak immunostaining (p<0.05). Surrounding normal thyroid tissue showed low HBME-1 levels only. CK-19 demonstrated distinct moderate-to-strong expression patterns in 100% of the tumor cells in cPTC cases as compared to benign samples (p<0.05), which were negative (Fig 3), and normal thyrocytes surrounding the cPTCs, which were also negative. S100A13 showed a weak up-regulation in cPTCs compared to benign cases (Fig 5), however the difference only showed borderline statistical significance (p = 0.07). Positive staining for S100A13 was observed in normal thyrocytes surrounding the cPTCs. Immunoexpression of ANXA3 and CMBL were detected in both cPTC and benign samples without being significantly different between the two groups. CMBL and ANXA3 exhibited similar immunostaining patterns in both cPTCs and benign samples, and positive staining for CMBL and ANXA3 was observed in normal thyrocytes surrounding the cPTCs (Fig 5).

**Table 3:** Summary of immunohistochemistry results of tissue samples from the cPTCs and benign cases.

Proteins	cPTC (n = 7)		Benign (n = 7)		cPTC vs. Benign p—value	Adjacent normal thyrocytes
	mean	(range)	mean	(range)		
HBME-1						
Intensity	2.2	(1–3)	1.0	(0–1)	**0.0001**	1
Positive cells	86%	(50–100%)	0.14%	(0–1%)	**0.001**	<1%
CK-19						
Intensity	2.3	(1.5–2.5)	1.0	(0–1)	**0.0008**	-
Positive cells	94.6%	(75–100%)	0.3%	(0–1%)	**0.001**	0
S100A13						
Intensity	3	(3–3)	2.4	(0–3)	0.07	2
Positive cells	100%	(100–100%)	86%	(0–100%)	0.7	100
ANXA3						
Intensity	2.4	(2–3)	2.3	(2–3)	0.5	2
Positive cells	100%	(100–100%)	100%	(100–100%)	1	100
CMBL						
Intensity	1.6	(1–2.5)	1.8	(1–2.5)	0.6	3
Positive cells	96.4%	(75–100%)	100%	(100–100%)	0.7	100

Intensity was scored as 0 = no staining; 1 = weak; 1.5 = weak to moderate; 2 = moderate; 2.5 = moderate to strong; 3 = strong. Statistically significant p-values are indicated in bold.

### Up-regulation of CK-19 and S100A13 in cPTCs

Based on the results from IHC and Western blot analyses, CK-19 and S100A13 were further analyzed by ELISA in non-depleted cyst fluid samples of the extended material of 17 cPTCs and 55 benign cystic lesions as well as reference samples (Table 4). We also included vimentin (VIM), which was identified as a down-regulated protein in cPTC by univariate analysis and in the multivariate OPLS model (S4 and S6 Tables).

**Table 4:** Summary of CK-19, S100A13 and vimentin concentrations by ELISA.

Parameter	CK-19 (CYFRA 21–1)	S100A13	Vimentin
***Protein concentrations by ELISA***			
cPTC: mean (range)	59 ng/ml (35–84)	244 pg/ml (229.3–267)	55 ng/ml (40–228)
Benign cystic lesions: mean (range)	36 ng/ml (1.5–71)	230 pg/ml (105–259)	62 ng/ml (35–147)
***Protein concentrations by ELISA in references***			
cPTC-Met-1	62 ng/ml	251 pg/ml	56 ng/ml
cPTC-Met-2	61 ng/ml	250 pg/ml	87 ng/ml
FTC-1	70 ng/ml	-	-
FTC-2	50 ng/ml	245 pg/ml	111 ng/ml
ATC	3 ng/ml	244 pg/ml	109 ng/ml
FToxA-1	35 ng/ml	249 pg/ml	56 ng/ml
FToxA-2	51 ng/ml	248 pg/ml	87 ng/ml
PHPT-1	60 ng/ml	253 pg/ml	83 ng/ml
***cPTC vs*. *Benign***			
p-value	**<0.001**	**0.021**	0.4
Direction of regulation	↑	↑	-
***Receiver operating characteristics (ROC)***			
Cut-off	55 ng/ml	230 pg/ml	-
Sensitivity	82% (57 to 96)	94%	-
Specificity	82%	35%	-
Positive predictive value (PPV)	82%	59%	-
Negative predictive value (NPV)	82%	86%	-
Positive likelihood ratio (PLR)	4.53	1.44	-
Negative likelihood (NLR)	0.22	0.17	-
Area under ROC curve (AUC)	0.86	0.69	-
Diagnostic accuracy	Good	Poor	-
Diagnostic odds ratio (DOR)	21	8.5	-

Statistically significant p-values are indicated in bold;— = not applicable or not available.

**Fig 6:**
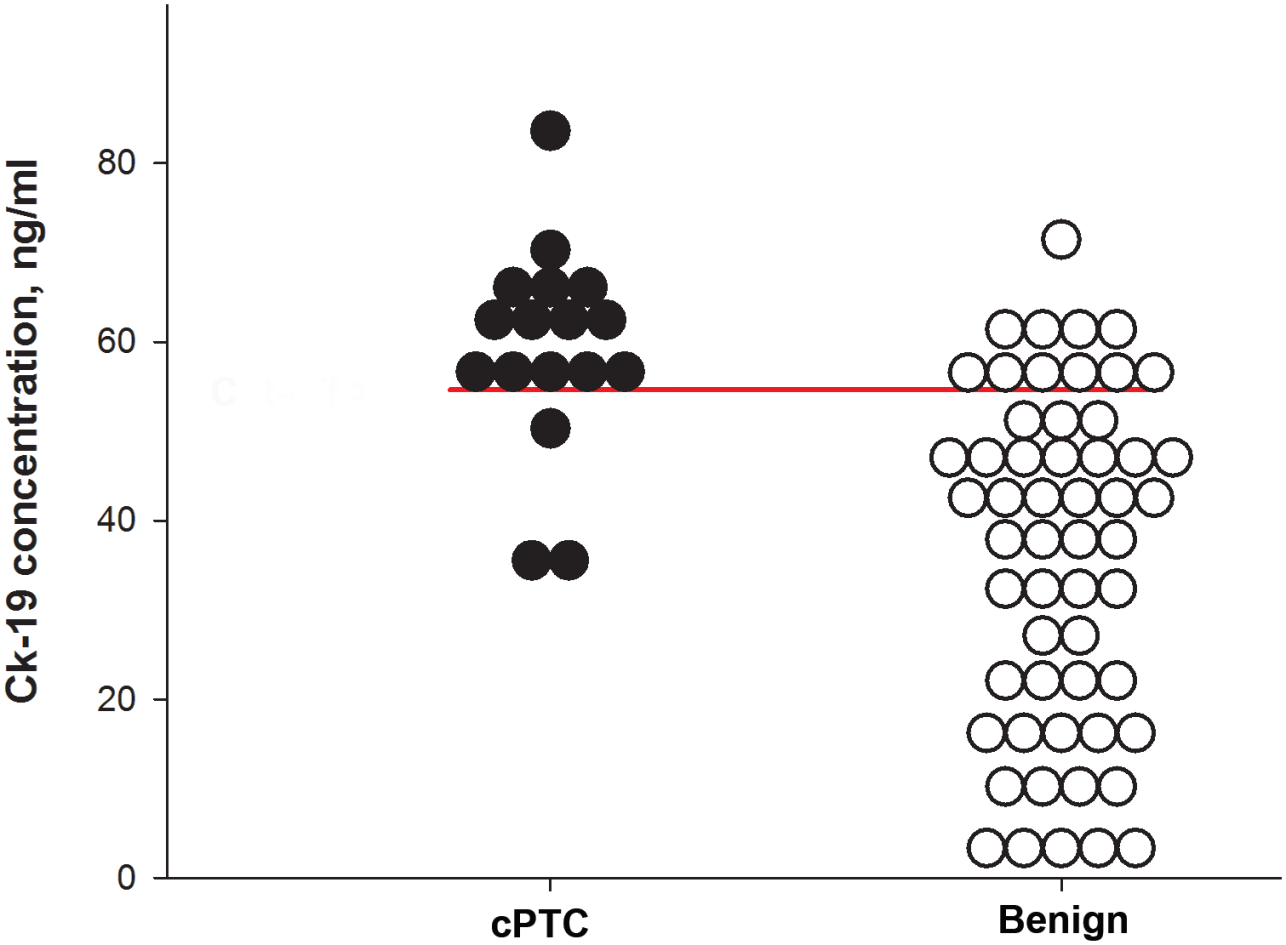
Scatter plots illustrating the distribution of CK-19 concentrations in fluid from cPTC and benign controls. The horizontal bar indicates the optimal cut-off for discrimination between cPTC and benign thyroid cyst samples.

The concentration of CK-19 was analyzed by applying the CYFRA 21–1 assay, detecting soluble fragments of CK-19. We found significantly higher concentrations of CK-19 in cyst fluids from cPTCs (mean 59 ng/ml) as compared to benign lesions (mean 36 ng/ml) (p<0.001), which is in agreement with the findings in the other three analyses (LC-MS/MS, Western blot and IHC). To determine the cut-off values we applied ROC curve analysis to the ELISA data and identified 55 ng/ml or more as the optimal cut-off value for CK-19 (Fig 6). At this cut-off, we obtained sensitivity and specificity of 82%, PPV and NPV of 82%, PLR of 4.53 and NLR of 0.22, which is in agreement with AUC (0.86), indicating good diagnostic accuracy of the ELISA test for CK-19/CYFRA 21–1 (p<0.0001) (Table 4). DOR was 21, meaning that the odds for diagnosis of cPTC are 21 times higher than the odds for patients with positive levels of CK-19 in cyst fluid of benign lesions.

S100A13 showed significant up-regulation in cPTCs (mean 244 pg/ml) as compared to benign lesions (mean 230 pg/ml), which is consistent with the LC-MS/MS data and partially with Western blot findings (p<0.05). ROC curve analyses of ELISA data revealed 230 pg/ml or more as an optimal cut-off for S100A13, showing sensitivity of 94%, specificity of 35%, PPV of 59%, NPV of 86%, PLR of 1.44 and NLR of 0.17, which is consistent with AUC (0.69) data indicating poor diagnostic accuracy (p = 0.02) (Table 4). Furthermore, DOR was 8.5. The concentration of VIM was not found to be significantly different between cPTCs and benign lesions (Table 4).

## Discussion

In this study we aimed to identify diagnostic markers, which can be applied additionally to FNAB, for discrimination between cPTC and benign thyroid cystic lesions through the investigation of the fluid accumulated by these lesions. Although the FNAB plays a major role in the differential diagnosis of thyroid lesions with an overall sensitivity level above 90%, this diagnostic tool is not always informative for cystic thyroid lesions [4, 6, 22]. The investigation of the liquid component of FNAB in such cases can potentially be performed for diagnostic purposes as suggested in studies of biochemical profiling of thyroid cyst fluid [5, 32] as well as supported by the results of proteomic profiling of cyst fluid from ovarian cancer and hemangioblastoma [22, 23]. The clinicopathological findings of the cPTCs in our study were consistent with studies of larger cohorts of cPTC and benign cysts reporting a majority of female patients aged >45 years [3, 4]. Similar to de los Santos *et al*, we observed differences in the appearance of cyst fluids suggesting differences in the proteome [4].

The proteins identified by LC-MS/MS were compared with a study evaluating deregulated genes on the mRNA level [33]. Totally 17 of the proteins identified in our dataset were also reported as consistently deregulated genes in PTC (S7 Table), supporting their possible relevance for PTC.

We investigated cyst fluids from benign thyroid lesions and cPTCs through global protein profiling using LC-MS/MS. Prior to LC-MS/MS, depletion of high abundant proteins was performed, to reduce the complexity of the samples and increase the chance of finding proteins specific to cPTC. Considering protein level patterns and the significance levels from the LC-MS/MS data as well as availability of antibodies, we selected CMBL, ANXA3, CK-19 and S100A13 for validation by IHC and WB in the same cases as used for LC-MS/MS screening. Further evaluation of CK-19, S100A13 and VIM was performed by ELISA using non-depleted cyst fluid from the extended material of cPTC and benign cystic thyroid lesions as well as cystic reference cases.

CK-19 is an epithelial cytoskeletal protein associated with classical PTC based on its frequent over-expression [6, 8]. However, CK-19 has not previously been analyzed in cyst fluids of thyroid lesions. We observed significantly up-regulated levels of CK-19 in depleted cyst fluids from cPTCs by LC-MS/MS, which was confirmed by IHC and WB. However, microscopical evaluation of morphologically normal thyrocytes surrounding cPTC did not show any CK-19 immunostaining, indicating a primary role of the cPTC cells in secretion of CK-19, which is also consistent with data from other publications [6, 8]. The staining patterns for HBME-1 were similar to CK-19, supporting the role of these proteins as post-operative markers of both classical and cystic PTC in clinical routine at the stage of final pathological diagnosis [5, 7, 8]. Further validation in the extended material was performed on non-depleted cyst fluid samples by ELISA, which is a commonly used method in clinical diagnostics. We used the CYFRA 21–1 assay to determine concentration of CK-19 in biological fluids, which is consistent with other reports investigating the diagnostic utility of CK-19 measurement in serum samples of patients with thyroid or lung cancers by analyses of cytokeratin fragments [34–36]. Our findings by ELISA demonstrate up-regulation of CK-19 in cPTCs, supporting the results of LC-MS/MS, IHC and WB experiments. By ROC curve analyses, we determined that 55 ng/ml or more is an optimal cut-off for CK-19 concentration in non-depleted cyst fluid for discrimination of cPTC from benign thyroid cysts by ELISA. Moreover, this suggested cut-off value is consistent with ROC curve parameters corresponding to a level of good diagnostic accuracy, supporting the role of CK-19 in diagnosis of cPTC. However, CK-19 concentrations >55 ng/ml were also found in cyst fluid from some benign thyroid samples, indicating a limitation for CK-19 application as an independent test (Table 4). Taken together, our data indicate that the determination of CK-19 concentration in cyst fluid by the CYFRA 21–1 assay can be an effective complementary tool to routine FNAB for discrimination of cPTC from benign thyroid cysts, which is in agreement with meta-analysis data suggesting CK-19 as a marker of thyroid malignancy [8].

S100A13 is a member of the calcium-binding S100 protein family. Ridinger *et al*. demonstrated IHC expression of S100A13 in normal thyroid tissue and cell-lines, whereas Cao *et al*. suggested this protein to promote proliferation in thyroid cancer cell-line [21, 37]. We identified a significant over-expression of S100A13 in cPTCs by LC-MS/MS, ELISA, partially in WB experiments, but IHC showed very similar expression patterns in cPTCs and benign lesions with only a slight increase and borderline significance. Furthermore, over-expression of S100A13 in cPTC samples indicates an association of this protein with cPTC, which is in agreement with results from other publications demonstrating up-regulation of S100 family members in primary PTC or its metastases to lymph nodes [12, 38]. To further evaluate our findings, we performed ROC curve analyses and determined that the concentration >230 pg/ml was the optimal cut-off for S100A13 in cyst fluid for discrimination of cPTC from benign thyroid cysts by ELISA. However, the analyses of ROC curve data revealed poor diagnostic utility of S100A13 by ELISA for diagnosis of cPTC. It is worth to mention that S100A13 has not been investigated neither in studies of thyroid cyst fluid nor in classical PTC proteome, therefore further evaluation in other cohorts may eventually justify the role of S100A13 in distinction between cPTCs and benign thyroid cystic lesions.

CMBL is a bioactivating hydrolase with high expression in the human liver and kidney [39]. We identified significantly different protein levels of CMBL in cyst fluid of cPTCs as compared to benign lesions by LC-MS/MS, however, IHC and WB showed similar expression patterns. It is worth to note that little is known about the biological role of CMBL in normal cells, while carboxymethylenebutenolidase recently was demonstrated in connection to hepatocellular carcinoma [18].

ANXA3 (also known as lipocortin 3) is a calcium-dependent phospholipid-binding protein in the annexin protein family showing over-expression in ovarian, lung, liver, colorectal and pancreatic cancers [40]. We demonstrated over-expression of ANXA3 in cPTCs as compared to benign lesions by LC-MS/MS. However, IHC and WB showed similar staining of ANXA3 in all cPTCs, including normal thyroid cells surrounding the cancer, which can be attributed to the diverse biological functions of ANXA3 in normal thyroid cells. In addition, our results are contradictory to the results from a previous proteomic study showing significant down-regulation of ANXA3 in classic PTC as compared to normal thyroid [41], and can indicate different pathophysiological properties between the cystic thyroid lesions and classical PTC.

VIM is an epithelial cytoskeletal protein associated with the epithelial-mesenchymal transition process and it is up-regulated in sarcoma [42]. We demonstrated down-regulation of VIM in cPTC by LC MS/MS, which is consistent with San Martin *et al*., who showed over-expression of VIM in a thyroid tissue microarray [43]. However, LC-MS/MS data was not confirmed by ELISA demonstrating similar concentration of VIM in cyst fluid from cPTC and benign samples. This finding is in agreement with Mato *et al*. who showed down-regulation of VIM in PTC cell-line, but controversial with Viale and co-workers who demonstrated expression of VIM in both PTC and benign lesions [44, 45].

## Conclusions

To summarize, we report protein profiles differing between benign cystic thyroid lesions and cPTCs based on proteomic analyses of cyst fluids. The findings may potentially lead to improved overall sensitivity of FNAB for thyroid cysts. Moreover, to the best of our knowledge, this is the first comprehensive catalogue of the protein content in fluid from thyroid cystic lesions. Based on data from LC-MS/MS, IHC, WB and ELISA we hypothesize that CK-19 could be determined in cyst fluid by ELISA and potentially used as a diagnostic marker of cPTC complementary to FNAB. The role of S100A13 and CK-19 proteins should be evaluated in prospective studies on independent tumor material using FNAB samples taken preoperatively.

## Supporting Information

S1 Fig Graphical illustration of all significantly enriched gene ontology (GO) terms and their internal relationships for the 841 overlapped proteins identified in cyst fluids. Each GO term is represented by a colored circle: the similar size indicates p<0.01 for all GO terms and the color intensity indicates the frequency of GO terms. Connected circles demonstrate highly related GO terms, and the width of the connecting lines is positively correlated to the degree of similarity.
(PPTX)

S2 Fig Comparison of proteomic data obtained for cyst fluids in the present study and a plasma reference data set published by Farrah T, Deutsch EW, Omenn GS, Campbell DS, Sun Z, Bletz JA, Mallick P, Katz JE, Malmström J, Ossola R, Watts JD, Lin B, Zhang H, Moritz RL, Aebersold R (2011). Molecular and Cellular Proteomics 10, M110.006353. (A) Venn diagram showing overlap between the plasma and the cyst fluid datasets. (B) Comparison of GO terms between plasma (blue) and cyst fluid (yellow).
(TIF)

S1 Table Clinical and histopathological features of cPTCs and benign cases included in the LC MS/MS experiments. (XLSX)

S2 Table Clinical and histopathological features of the cystic tumors used as controls. (XLSX)

S3 Table Details of iTRAQ labeling and fluid properties of depleted fluid samples used in LC MS/MS. (XLSX)

S4 Table Overlapping proteins identified in cyst fluids from cPTCs and benign cystic thyroid lesions. (XLSX)

S5 Table Summary of gene ontology analyses of 841 proteins identified by LC MS/MS in depleted cyst fluid. (XLSX)

S6 Table Orthogonal Partial Least Squares (OPLS) predictive model of 59 proteins differently expressed in cPTCs vs. benign cyst fluids. (XLSX)

S7 Table Proteins identified by LC-MS/MS with consistently deregulated corresponding gene expression in PTC. (XLSX)
