# Supplementary material for: Differential Protein Expression Profiles of Cyst Fluid from Papillary Thyroid Carcinoma and Benign Thyroid Lesions
Source: PLoS One. 2015 May 15;10(5):e0126472. doi: 10.1371/journal.pone.0126472 (PMC4433121; doi:10.1371/journal.pone.0126472)
Supplement: S1 Fig — Each GO term is represented by a colored circle: the similar size indicates p<0.01 for all GO terms and the color intensity indicates the frequency of GO terms. Connected circles demonstrate highly related GO terms, and the width of the connecting lines is positively correlated to the degree of similarity. (PPTX) [file pone.0126472.s001.pptx]

## Slide 1
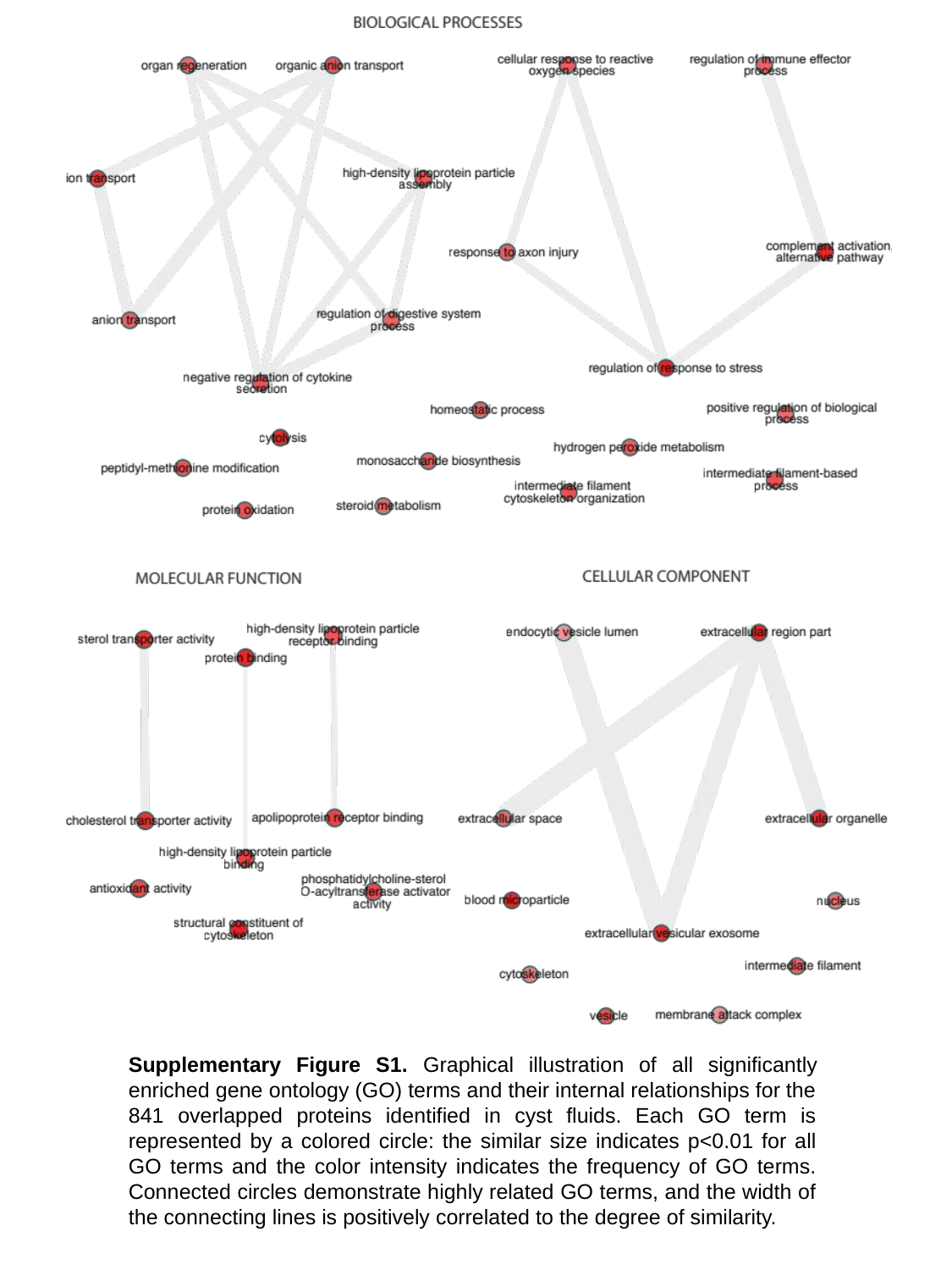

Supplementary Figure S1. Graphical illustration of all significantly enriched gene ontology (GO) terms and their internal relationships for the 841 overlapped proteins identified in cyst fluids. Each GO term is represented by a colored circle: the similar size indicates p<0.01 for all GO terms and the color intensity indicates the frequency of GO terms. Connected circles demonstrate highly related GO terms, and the width of the connecting lines is positively correlated to the degree of similarity.
