# Supplementary figures and images for: Differential Protein Expression Profiles of Cyst Fluid from Papillary Thyroid Carcinoma and Benign Thyroid Lesions
Source: PLoS One. 2015 May 15;10(5):e0126472. doi: 10.1371/journal.pone.0126472 (PMC4433121; doi:10.1371/journal.pone.0126472)

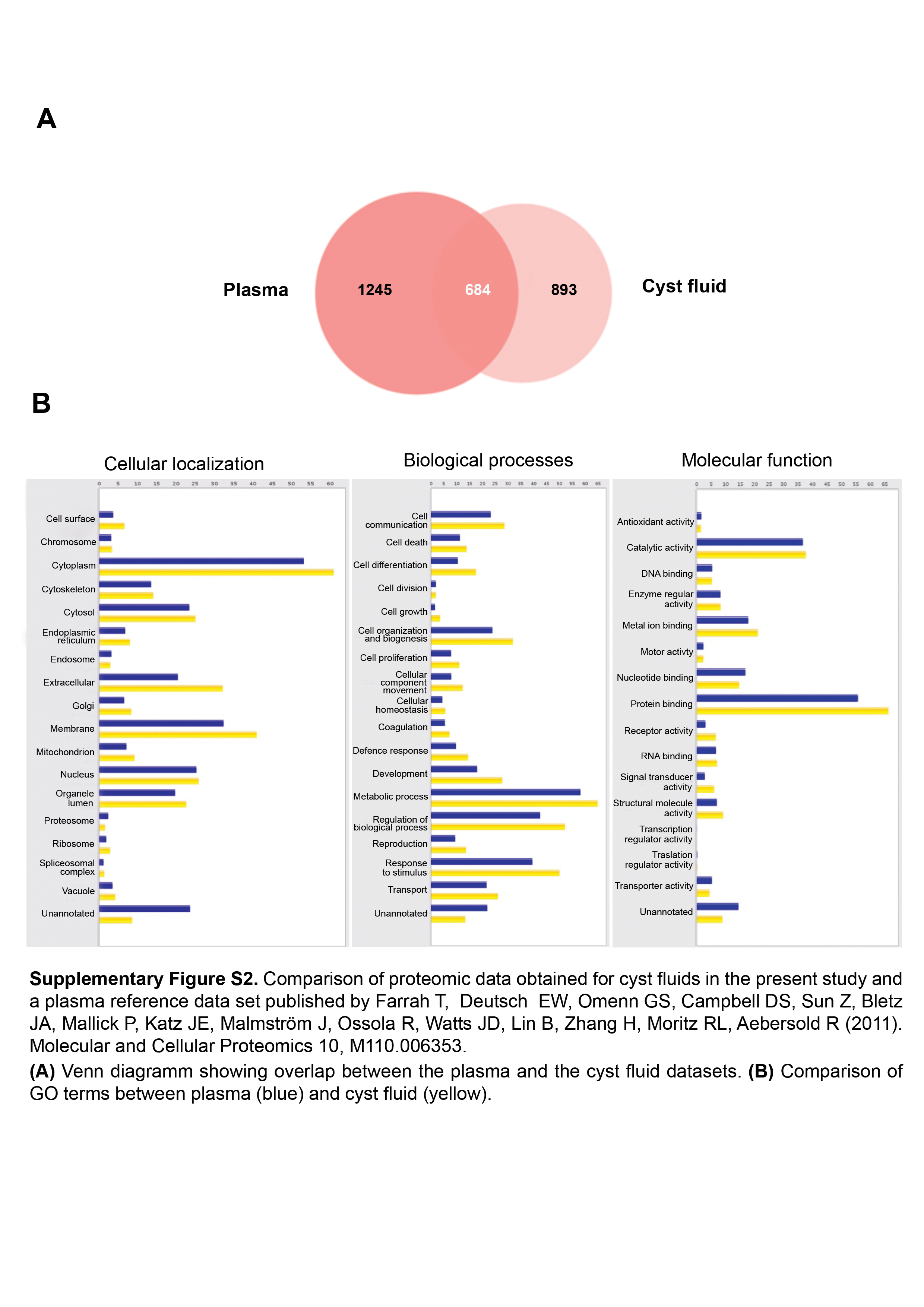

Supplement: S2 Fig — (A) Venn diagram showing overlap between the plasma and the cyst fluid datasets. (B) Comparison of GO terms between plasma (blue) and cyst fluid (yellow). (TIF) [file pone.0126472.s002.tif]
